# Acute Jejunal Diverticulitis: A Case Report

**DOI:** 10.7759/cureus.101609

**Published:** 2026-01-15

**Authors:** Rebecca Sedivy, Anmol Nigam, Pravin Meshram, Rubeena Naaz, Chidiebere Onongaya, Byoung Uk Park, Sean H Nguyen, James V Harmon, John McCormick-Deaton

**Affiliations:** 1 Department of Surgery, University of Minnesota, Minneapolis, USA; 2 Department of Surgery, Shadan Institute of Medical Sciences, Hyderabad, IND; 3 Department of Laboratory Medicine and Pathology, University of Minnesota, Minneapolis, USA

**Keywords:** acute abdomen, case report, ileum, jejunal diverticulitis, jejunum, perforated jejunal diverticulitis, small intestinal diverticulitis

## Abstract

Acquired small intestinal diverticulitis and its complications remain poorly understood due to their rarity and nonspecific presentation. Complications such as abdominal sepsis, bleeding, and perforation can lead to unfavorable outcomes. We report the case of a patient who presented with acute jejunal diverticulitis requiring surgical intervention.

A 66-year-old man with a history of endovascular abdominal aortic aneurysm repair presented with acute left lower quadrant abdominal pain and tenderness. Computed tomography (CT) detected intestinal diverticulosis, localized free air, and inflammatory changes associated with the small intestine. Exploratory laparoscopy revealed numerous proximal jejunal diverticula with perforation contained within the mesentery. A 48 cm segment of the jejunum was resected, and a primary anastomosis was performed. Surgical pathology confirmed acute small intestinal diverticulitis and serositis. The patient had an uneventful postoperative course and was discharged home on postoperative day 6.

This case highlights the diagnostic challenge in acute small intestinal diverticulitis, which may present with nonspecific signs and inconclusive radiological findings. The location of diverticula along the mesenteric border may contain perforations in the mesentery and obscure peritoneal signs. Although some patients with acute small intestinal diverticulitis may be conservatively managed, surgical intervention remains the standard of care for managing patients with complications.

Acute small intestinal diverticulitis, though exceedingly rare, can carry a significant risk of morbidity and mortality. Thus, small intestinal diverticulitis should be considered in the differential diagnosis in older adults who present with acute abdominal pain and tenderness. Surgical management remains the gold standard of treatment of severe acute small intestinal diverticulitis.

## Introduction

Acute small intestinal diverticulitis is a rare condition in which small pouches in the small intestine wall (diverticula) become inflamed. These diverticula are considered pseudodiverticula, consisting of mucosal, submucosal, and serosal layers without muscular involvement. They arise at weak points where the vasa recta penetrate the muscularis propria along the mesenteric border [1]. This anatomic location is clinically significant as the mesentery can obscure diverticula on imaging. When perforation occurs, it may be contained within the mesentery, attenuating physical examination findings and masking disease severity [2,3].

Small intestinal diverticulosis has an estimated incidence of 0.06-2.3% and most commonly affects men in their sixth and seventh decades of life [2-8]. Although most cases remain asymptomatic, 10-20% develop complications such as acute diverticulitis, hemorrhage, intestinal and biliary obstruction, and perforation [2-8]. Perforation occurs in 2.1-7% of jejunal diverticulitis cases and carries a mortality rate of up to 40% [9,10]. Despite these significant outcomes, the rarity of the condition limits available evidence to case reports and small case series; no standardized guidelines for management exist [2,11].

Here, we report a case of perforated jejunal diverticulitis, wherein the perforation was contained within the mesentery and the patient lacked peritoneal signs. This case underscores the diagnostic challenge posed by attenuated clinical findings and supports early operative intervention as a safe and effective approach even when clinical presentation might otherwise favor conservative management.

## Case presentation

A 66-year-old man presented to the emergency department with a sudden onset of severe left lower quadrant abdominal pain that awakened him from sleep. He reported no prior symptoms, nausea, vomiting, or changes in his bowel habits. His past medical history included endovascular repair of an infrarenal abdominal aortic aneurysm (AAA) six months prior to presentation, a known type II endoleak, remote laparoscopic cholecystectomy, and open abdominal exploration to repair an incarcerated incisional hernia with small intestinal obstruction that did not require resection.

Upon presentation, the patient was hypertensive with a blood pressure of 160/92 mmHg; other vital signs were within normal limits. Physical examination revealed increased work of breathing and focal left-sided abdominal tenderness. Notably, his abdomen was soft and nonperitoneal. Laboratory test results revealed leukocytosis with a white blood cell (WBC) count of 15.7×103/uL.

**Figure 1:**
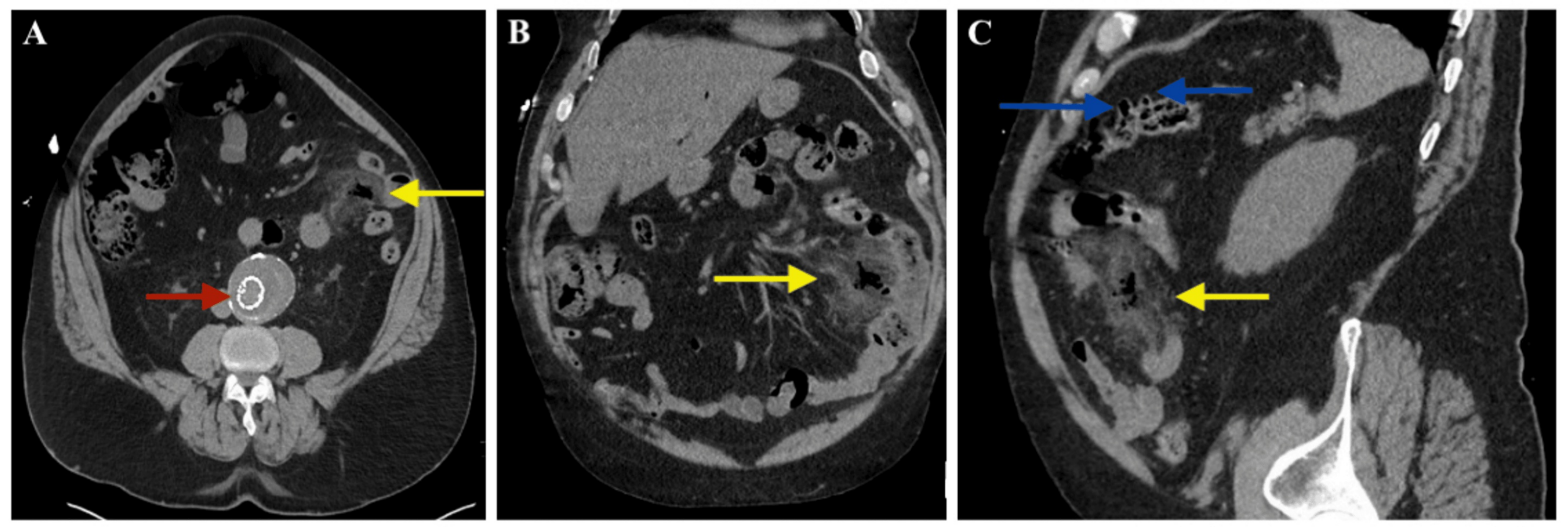
Abdominal computed tomography imaging Mesenteric inflammation and localized intramesenteric free air are indicated by yellow arrows (A-C). The stable abdominal aortic aneurysm repair is marked with a red arrow. Two small intestinal diverticula are visible (C, blue arrows).

Given the patient's recent AAA repair and known endoleak, computed tomography (CT) angiography of the chest, abdomen, and pelvis was performed. Imaging demonstrated a stable AAA repair and sigmoid diverticulosis. However, it also revealed free air and inflammatory changes localized to the left lower quadrant that appeared to be associated with the small intestine rather than the sigmoid colon (Figure 1). The perforation appeared contained within the mesentery; however, its unclear etiology and proximity to the aortic graft raised concerns. Emergent exploratory laparoscopy was performed for diagnostic clarification and definitive management.

**Figure 2:**
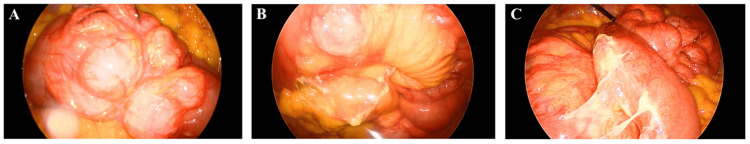
Intraoperative findings (A) Multiple small intestinal diverticula along the mesenteric border. (B,C) Suppurative inflammation, adhesions, and gross perforation of the involved jejunal segment.

Intraoperatively, the small intestine was examined from the terminal ileum to the ligament of Treitz. Purulent fluid was noted in the left upper quadrant; a segment of the proximal jejunum near the ligament of Treitz with numerous diverticula was identified. The mesentery was adherent to the abdominal wall, and dissection revealed a mesenteric abscess with a single perforated diverticulum as the source (Figure 2).

**Figure 3:**
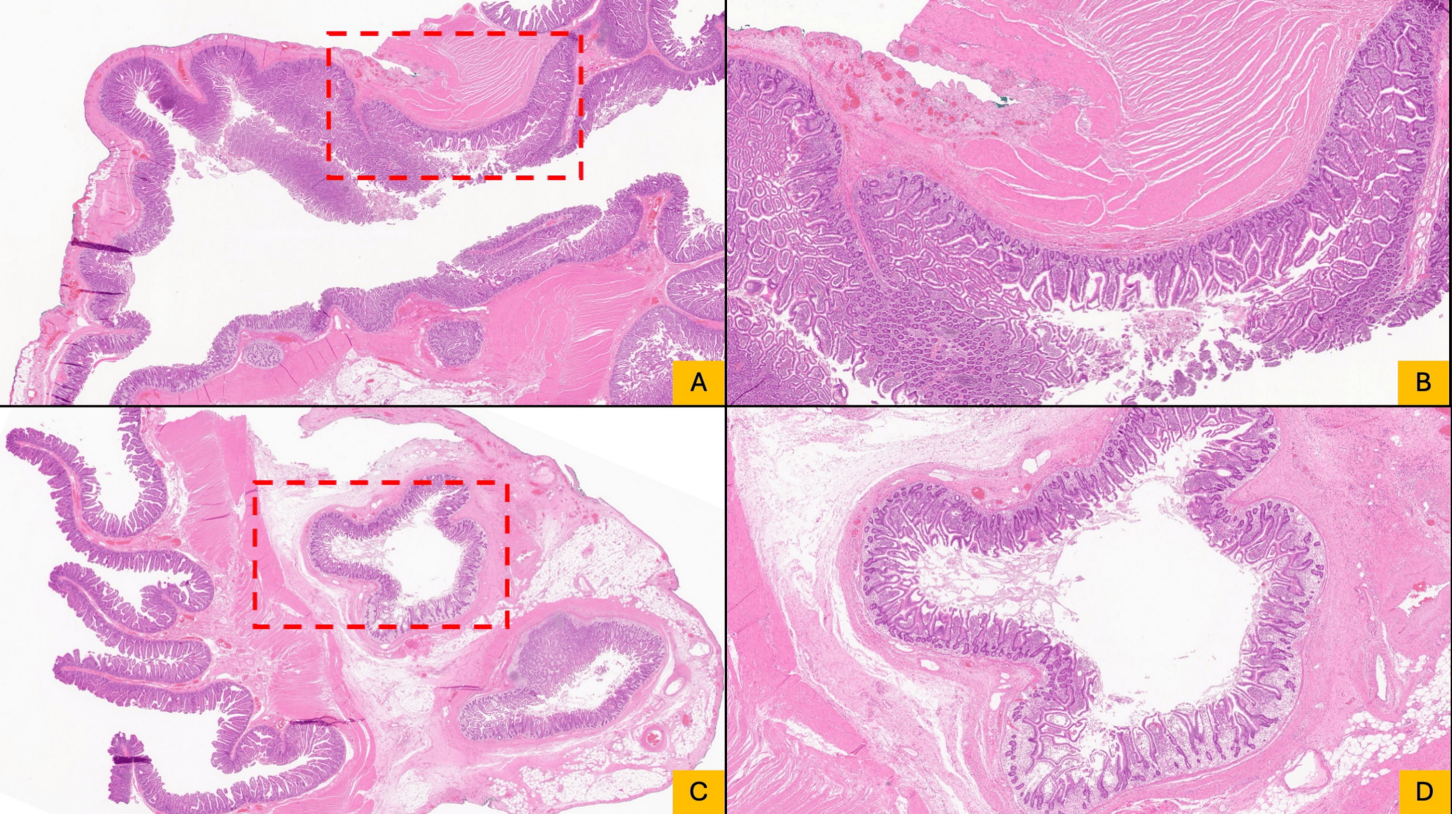
Histologic sections of jejunal diverticula Longitudinal (A, B) and cross-sectional (C, D) views demonstrating the outpouching of the mucosa and submucosa through the muscularis propria without complete muscular investment, consistent with pseudodiverticula. Panels A and C are low magnification (1.5×) with higher-magnification views (4×) shown in B and D.

A 48 cm segment of the jejunum was resected with primary stapled anastomosis. Surgical pathology confirmed acute small intestinal diverticulitis with serositis (Figure 3).

The patient recovered well postoperatively. He was started on a diet on postoperative day 3, completed a five-day course of antibiotics, and was discharged home on postoperative day 6. He remained well at the three-month follow-up.

## Discussion

We report a case of acute perforated jejunal diverticulitis in a 66-year-old man who presented with acute abdominal pain and leukocytosis but lacked peritoneal signs despite having a perforation. This case underscores the diagnostic challenges posed by the mesenteric location of small intestinal diverticula and supports early operative intervention as a safe and effective approach even when clinical findings might otherwise favor conservative management.

Small intestinal diverticulitis most commonly affects men in their sixth and seventh decades of life [4,5]. In cases of perforation, symptoms typically include acute abdominal pain, leukocytosis, nausea, and vomiting. However, signs of peritonitis, such as guarding and rebound tenderness, might be absent. In one case series, only three of 13 patients with jejunal diverticular perforations were peritonitic on examination [12]. Our patient presented with acute abdominal pain and leukocytosis but without nausea, vomiting, or peritoneal signs. This relatively benign presentation despite perforation is explained by the mesenteric location of small intestinal diverticula, which allows the mesentery to contain perforations and attenuate physical examination findings. This discordance between clinical presentation and underlying pathology underscores the importance of prompt imaging in older patients presenting with acute abdominal pain and leukocytosis, even when examination findings are reassuring.

According to the American College of Radiology, contrast-enhanced CT of the abdomen and pelvis is the optimal imaging modality in patients exhibiting left lower quadrant pain [13]. In this case, CT angiography was appropriate given the patient's recent endovascular aneurysm repair and initial concern about graft-related complications. Imaging demonstrated sigmoid diverticulosis along with free air and inflammatory changes localized to the left lower quadrant. The radiology report noted that these findings appeared to be associated with the small intestine rather than the sigmoid colon. However, the jejunal diverticula themselves were not formally identified. This highlights the difficulty in recognizing small intestinal diverticula on imaging, particularly when sigmoid diverticulosis is also present, and offers a more common explanation for the findings. Definitive diagnosis often requires operative exploration.

Given the clinical and radiographic findings suggesting small bowel perforation, we proceeded with exploratory laparoscopy for diagnostic clarification and definitive management. A laparoscopic approach was selected since it has been associated with fewer complications and a shorter length of stay compared with open resection for diverticular disease [14,15]. After identifying the perforation site, a small midline incision was made to allow hand assistance while maintaining pneumoperitoneum, avoiding the need for full conversion to an open approach.

The optimal management of perforated small intestinal diverticulitis remains unclear, particularly when perforation is contained and the patient is hemodynamically stable. Some authors advocate for conservative management with bowel rest, gastric decompression, antibiotics, and drainage of fluid collections, extrapolating from the shift toward nonoperative management seen in colonic diverticulitis [16,17]. Our patient might have been a candidate for this approach given his contained perforation, stable vital signs, and lack of generalized peritonitis. In one reported case, a patient with similar features was successfully treated nonoperatively with a five-day course of intravenous antibiotics and bowel rest [18]. However, evidence supporting conservative management of perforated small intestinal diverticulitis is limited to isolated case reports, and multiple reports describe failure of nonoperative management with subsequent need for surgery [19-21]. Furthermore, delayed surgical intervention has been associated with increased contamination. One review found that patients who underwent surgery within 24 hours had mild contamination limited to the mesentery, whereas those with delays beyond 72 hours had severe, generalized contamination [12].

Another study adopted the modified Hinchey classification system for small intestinal diverticulitis and suggested that a stage III disease (generalized purulent peritonitis) requires surgical intervention [22]. However, optimal management of stage Ib (confined peri-intestinal abscess) and stage II (distant mesenteric abscess) remains undefined. Our patient, with a contained perforation and mesenteric abscess without generalized peritonitis, would fall into this intermediate category, further illustrating the management uncertainty that exists for this subset of patients.

In the absence of established guidelines, operative management is generally recommended for perforation with gross contamination [22]. However, in patients, such as ours, with contained perforations who are hemodynamically stable, the optimal approach is less clear. Given the potential for progression with delayed intervention and the difficulty in establishing a definitive diagnosis preoperatively, we pursued early operative management with a good outcome.

## Conclusions

Perforated small intestinal diverticulitis is rare but carries a significant mortality risk. The mesenteric location of small intestinal diverticula can contain perforations and attenuate clinical findings, creating a presentation that underrepresents disease severity. Clinicians should maintain a high index of suspicion in older patients presenting with acute abdominal pain and leukocytosis, even when peritoneal signs are absent. In the absence of consensus guidelines, this case supports early operative intervention as a safe and effective approach for perforated jejunal diverticulitis, even when clinical presentation might otherwise favor conservative management.
